# Graphene oxide/ε-poly-L-lysine self-assembled functionalized coatings improve the biocompatibility and antibacterial properties of titanium implants

**DOI:** 10.3389/fbioe.2024.1381685

**Published:** 2024-04-04

**Authors:** Xiaoxiao You, Zhongke Wang, Li Wang, Youbo Liu, Hongmei Chen, Xiaorong Lan, Ling Guo

**Affiliations:** ^1^ Department of Oral Prosthodontics, The Affiliated Stomatological Hospital, Southwest Medical University, Luzhou, China; ^2^ Institute of Stomatology, Southwest Medical University, Luzhou, China; ^3^ School of Stomatology, Southwest Medical University, Luzhou, China; ^4^ Luzhou Key Laboratory of Oral & Maxillofacial Reconstruction and Regeneration, Luzhou, China; ^5^ The Public Platform of Zebrafish Technology, Public Center of Experimental Technology, Southwest Medical University, Luzhou, China

**Keywords:** graphene oxide, ε-poly-L-lysine, antibacterial, biocompatibility, layer-by-layer self-assembly

## Abstract

The construction of an antibacterial biological coating on titanium surface plays an important role in the long-term stability of oral implant restoration. Graphene oxide (GO) has been widely studied because of its excellent antibacterial properties and osteogenic activity. However, striking a balance between its biological toxicity and antibacterial properties remains a significant challenge with GO. ε-poly-L-lysine (PLL) has broad-spectrum antibacterial activity and ultra-high safety performance. Using Layer-by-layer self-assembly technology (LBL), different layers of PLL/GO coatings and GO self-assembly coatings were assembled on the surface of titanium sheet. The materials were characterized using scanning electron microscope (SEM), Fourier transform infrared spectroscopy (FTIR), X-ray photoelectron spectroscopy (XPS) and contact angle test. The antibacterial properties of Porphyromonas gingivalis (*P.g*.) were analyzed through SEM, coated plate experiment, and inhibition zone experiment. CCK-8 was used to determine the cytotoxicity of the material to MC3T3 cells, and zebrafish larvae and embryos were used to determine the developmental toxicity and inflammatory effects of the material. The results show that the combined assembly of 20 layers of GO and PLL exhibits good antibacterial properties and no biological toxicity, suggesting a potential application for a titanium-based implant modification scheme.

## 1 Introduction

Implant restoration has become a crucial method for replacing missing teeth due to its excellent function and aesthetic effects (Jiang et al., 2020; Li et al., 2022b). However, 42%–43% patients will suffer from peri-implant mucositis after oral implant restoration, and 20%–22% patients will suffer from peri-implantitis (Lee et al., 2017; Onclin et al., 2022; Suzuki et al., 2023). What is more concerning is that peri-implant mucositis can further develop into peri-implantitis (Herrera et al., 2023). Peri-implantitis is a progressive inflammatory disease that causes irreversible damage to the soft and hard tissues around the implant, threatening the long-term survival of implants (Schwarz et al., 2018; Cai et al., 2019). Research has shown that the accumulation and colonization of bacterial biofilms around implants are an important cause of peri-implant inflammation (Cai et al., 2019; Qian et al., 2020), and once colonized, bacteria become difficult to remove (Yu et al., 2022). The long-term survival of implants requires that implant materials possess excellent osteogenesis, antibacterial properties, and biocompatibility (Shao et al., 2022). While titanium is widely used in the production of dental implants (Souza et al., 2019), it lacks osteogenic and antibacterial properties (Ji et al., 2023). Therefore, an increasing number of researchers are beginning to modify the implant surface to prevent peri-implant diseases.

Graphene oxide (GO) is a new type of two-dimensional carbon material that holds significant potential in the field of biomedicine (Yousefi et al., 2017; Gusev et al., 2019). GO possesses a unique amphiphilic structure that can interact with cells or growth/differentiation factors, accelerating the adhesion, growth and differentiation of bone marrow stem cells (Wei et al., 2017), neural stem cells (Lin et al., 2020), and induced pluripotent stem cells (Saburi et al., 2019; Ricci et al., 2022). Studies have reported that when GO is used as a scaffold or coating material, it can enhance the regeneration of bone and cartilage (Li et al., 2022b). Additionally, direct contact of GO with bacteria can activate mechanisms such as nano-knife (Sun et al., 2020), encapsulation or capture (Du et al., 2022), oxidative stress (Liu et al., 2011), and has an evident antibacterial effect on both Gram-negative and Gram-positive bacteria (Pulingam et al., 2019)^.^ However, the inhibitory ability of GO to planktonic bacteria around the implant is limited (Pandit et al., 2023). Furthermore, the antibacterial activity of GO is concentration-dependent, meaning that at high concentrations, GO may induce biotoxicity. To address the issue of low bactericidal efficiency of GO with non-biotoxic concentrations, consideration was given to loading ε-poly-L-lysine (PLL) onto the titanium surface.

ε-poly-L-lysine (PLL) is a cationic polypeptide polymerized from 25–35 L-lysine (Wang et al., 2021). Presently, it finds extensive applications in the fields of food preservation and medicine (Zhang et al., 2018a). PLL exhibits high antibacterial activity and broad-spectrum effects. Studies have confirmed its efficacy against various Gram-negative bacteria, Gram-positive bacteria, fungi, and viruses (Bankar and Singhal, 2010). Additionally, PLL possesses water solubility, biodegradability, and shows no human or environmental toxicity, demonstrates extremely high safety (Wang et al., 2021). Consequently, PLL is considered to be a safe agent by the United States Food and Drug Administration, and is approved for food preservation in the United States, China, Japan, and other countries (Zhang et al., 2018a; Li et al., 2022a).

To effectively control the deposition amount of PLL and GO, layer-by-layer self-assembly technology (LBL) demonstrates significant technical advantages due to its simple process and mild preparation conditions (Zhang et al., 2018b). LBL utilizes electrostatic interactions to alternately adsorb positively/negatively charged polyelectrolytes on the surface of materials, forming composite multilayer films where the biological activities of the components do not interfere with each other (Lv et al., 2014; Alkekhia et al., 2020). Moreover, LBL offers the advantage of accurately controlling the thickness of the film on a nano-scale and can delay the release of drugs while maintaining sustained antibacterial ability, making it a promising coating method (Gentile et al., 2015). Numerous studies have demonstrated that biologically active coatings containing GO, constructed using LBL techniques, exhibit significant effects in areas such as cardiovascular stents and bone implants. Research by Xu et al. (Xu et al., 2021) illustrates that polydopamine/GO/type I collagen nanofilms exhibit strong capabilities for controlled release of bioactive substances, enhancing not only protein adsorption but also promoting osteogenic differentiation of rat bone marrow-derived mesenchymal stem cells. Furthermore, studies indicate that the greater the number of nanofilm layers, the more pronounced the osteogenic differentiation effect (Xu et al., 2021). Studies by Gao et al. indicate that chitosan/heparinized GO multilayer films can promote adhesion and proliferation of endothelial cells, demonstrating good blood compatibility and corrosion resistance, thus providing new insights for the development of cardiovascular stent materials.

The objective of this study is to assemble PLL and GO on the surface of a titanium sheet to enhance antibacterial properties. The optimal number of assembly layers with good biocompatibility was determined through *in vivo* and *in vitro* experiments. This study introduces novel approaches to address peri-implant diseases and enhance the long-term retention of implants.

## 2 Materials and methods

### 2.1 Materials

PLL was procured from Shanghai Aladdin Biochemical Technology Co., Ltd. The sheet diameters of GO, carboxylated graphene oxide (GO-COOH) and aminated graphene oxide (GO-NH_2_) are all less than 500 nm and these materials were obtained from Jiangsu Xianfeng Nanomaterials Technology Co., Ltd. Titanium sheets (purity 99.99%) were supplied by Taizhou Senno Material Technology Co., Ltd. Preosteoblasts MC3T3-E1 used in the experiment were obtained from ATCC. Luzhou Keyang Biotechnology Co., Ltd. provided Porphyromonas gingivalis, as well as culture media and reagents for cell and bacterial experiments. The zebrafish and breeding materials used in the experiment were obtained from the zebrafish technology platform of the Public Experimental Technology Center of Southwest Medical University.

### 2.2 Fabrication of composite coatings on titanium surface

**FIGURE 1:**
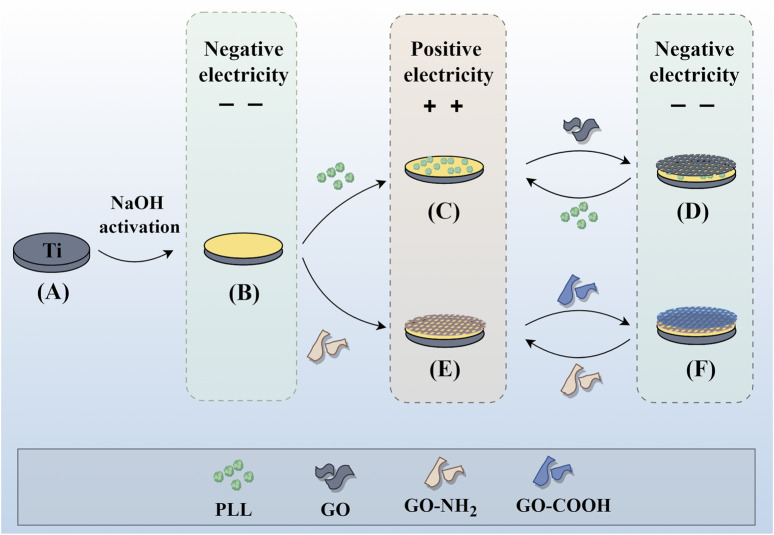
Individual assembly of GO and combined assembly of GO and PLL on the titanium surface. Assembly process of P/G and G/G on the titanium surface. **(A)** Pure titanium. **(B)** Titanium surface with negative charge after alkali activation. **(C)** Deposition of positively charged PLL. **(D)** Deposition of negatively charged GO. **(E)** Deposition of positively charged GO-NH_2_. **(F)** Deposition of negatively charged GO-COOH. Repeat (C) (D) or (E) (F) to achieve the desired number of layers.

Pure titanium sheets were processed into circular samples with a diameter of 15 mm and a thickness of 1 mm, and their surfaces were polished with #800, #1200, and #2000 silicon carbide paper under running water. Then, they were ultrasonically cleaned in acetone, ethanol, and deionized water for 15 min and dried at 60°C for 24 h. As shown in Figure 1 (Figure 1 was drawn by Figdraw), to obtain active titanium sheets (ATi), the samples were soaked in 5 M sodium hydroxide (NaOH) solution at 60°C for 24 h, then rinsed with distilled water 3 times and dried for use. Half of the samples were soaked in 1 mg/mL PLL for 10 min, rinsed with deionized water, followed by nitrogen drying. Subsequently, they were immersed in 1 mg/mL GO for 10 min, rinsed, and dried, thereby constructing a bilayer membrane structure. The above steps were repeated to obtain multilayer samples with different layers, labeled as (P/G)_10_, (P/G)_20_, (P/G)_30_. Similarly, the other half of the samples were alternately immersed in 1 mg/mL of GO-COOH and 1 mg/mL of GO-NH_2_, and the film assembly process was repeated to obtain (G/G)_10_, (G/G)_20_, (G/G)_30_.

### 2.3 Characterization of samples

**FIGURE 2:**
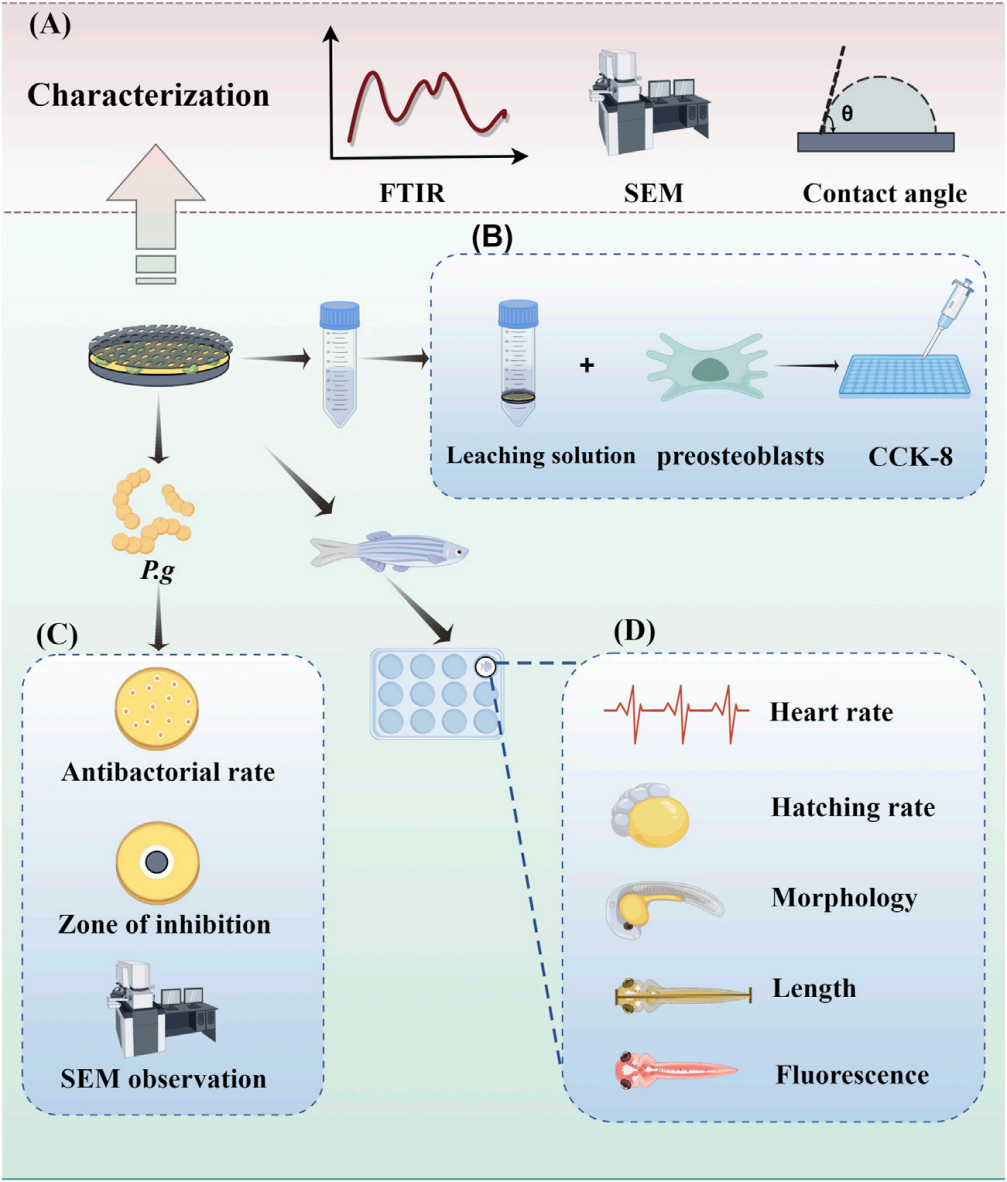
Experimental operation diagram. **(A)** Characterization of samples. **(B)** Evaluation of antibacterial ability. **(C)** Cytotoxicity assays. **(D)** *In vivo* toxicity assessment.

As shown in Figure 2A (Figure 2 was drawn by Figdraw), the surface morphology of the samples was characterized by scanning electron microscopy (SEM, Thermo Fisher, USA), and the chemical composition of the samples was collected and analyzed by energy dispersive X-ray spectroscopy (EDS, AMETEK,USA). The chemical composition of the modified surface was analyzed using the attenuated total reflection attachment (ATR) of Fourier transform infrared spectroscopy (FTIR, Beifen-Ruili, China) and X-ray photoelectron spectroscopy (XPS, Thermo Fisher ScientificK-Alpha, USA). Three samples were selected for each group, and 5 μL of deionized water droplets were placed on the surface of the sample at room temperature. The water contact angle was measured by contact angle measuring instrument (KRUSS, DSA25, Germany) and photographed.

### 2.4 Evaluation of antibacterial ability

#### 2.4.1 Observation of bacterial morphology

P.g was anaerobically cultured in BHI broth at 37°C to logarithmic growth stage, and the concentration of bacterial suspension was adjusted to 1.0 × 10^6^ CFU/mL for later use. Samples from each group were placed face up in a 12-well plate, 200 μL of bacterial suspension was applied to the surface of the sterilized sample and carefully transferred to an anaerobic incubator for 2 h of cultivation. After stable bacterial adhesion, 0.8 mL of BHI medium was added to each well for further cultivation. The sample was co-cultured with bacteria for 24 h, then, the surface of the sample was washed with aseptic PBS twice to remove unattached bacteria. Subsequently, 2.5% glutaraldehyde was fixed for 20 min, rinsed with PBS, followed by ethanol gradient dehydration and drying. The surface of the sample was sprayed with gold, and the microscopic morphology of *P.g* on the surface of each group was observed by SEM.

#### 2.4.2 Antibacterial rate

The bacterial suspension was co-cultured with the samples for 24 h. After washing off the suspended bacteria, the samples were placed in 1 mL PBS and vortexed for 10 min to collect the adhered bacteria on the surface of the sample. The concentration of bacterial suspension was adjusted to 1.0 × 10^3^ CFU/mL. A 100 μL bacterial suspension was evenly spread on BHI solid medium, and after anaerobic culture for 48 h, the antibacterial rate was calculated using the following formula.

$$A\text{ntibacterial rate}\left(\%\right)=\frac{CFU\;\left(Control\right)-CFU\;\left(\text{Experimental}\right)}{CFU\;\left(Control\right)}$$

#### 2.4.3 Inhibition zone test

A 100 μL bacterial suspension was uniformly inoculated on the surface of the BHI solid medium. After sterilization, each sample was carefully placed face down in BHI medium and anaerobically cultured for 48 h. The inhibition of each sample against the surrounding bacteria was recorded.

### 2.5 Cytotoxicity assays

MC3T3-E1 cells were incubated with α-MEM medium containing 10% fetal bovine serum (FBS), 100 U/mL penicillin and 100 ug/mL streptomycin in a 5% CO_2_ incubator at 37°C. The culture medium was changed every 2 days and passaged at a ratio of 1:3. After sterilization, the samples of each group were immersed in α-MEM medium for 1 week, and 1 mL of leaching solution was collected for each sample. The extraction medium was prepared with different leaching solutions instead of α-MEM and FBS in a 9:1 ratio. Well-grown cells were cleaned with PBS, digested by pancreatic enzymes, and then centrifuged to produce a 1×10^4^/mL cell suspension. A 100 μL cell suspension was inoculated into 96-well plates with three compound wells in each group. After culture for 24 h, the previous medium was discarded, and the prepared extraction medium was added. After another 24 h, 100 μL medium containing 10% CCK-8 was added to each well. After incubation for 2 h, the absorbance was determined at the wavelength of 450 nm using an enzyme labeling instrument.

### 2.6 *In vivo* toxicity assessment

The zebrafish involved in the experiment were sourced from the Zebrafish technology platform of the Public Experimental Technology Center of Southwest Medical University. Wild-type AB zebrafish were fed with newly hatched harvest insects twice a day in a zebrafish culture system with a constant temperature of 28°C and a light-dark photoperiod of 14:10 h. After reaching the adult stage, healthy male and female fish of similar size were selected for mating and egg laying. The fertilized eggs were sorted under the microscope (Nikon, Japan). Sodium hypochlorite disinfectant (0.003%) and sterilized embryo culture water containing 0.3 × Danien’s buffer were alternately added to the culture plate for 5 min each. The disinfectant should completely submerge the embryos, and the plate should be gently shaken to ensure full contact. Subsequently, the disinfectant was filtered, and sterilized embryo culture water was added and washed twice to ensure complete removal of sodium hypochlorite. Samples were placed in a 12-well plate, and embryos 6 h after fertilization were directly exposed to the surface of the test material. There were 10 embryos in each group, and the experiment was conducted twice. The embryos were incubated in a constant-temperature incubator. At 72 hpf, the incubation and mortality of the embryos were observed and recorded under a stereomicroscope. At 120 hpf, the heart rate, body length and developmental morphology of zebrafish were observed and recorded under 0.1% (w/v) tricaine solution anesthesia. After the experiment is completed, embryos are euthanized by freezing at −20°C for ≥24 h. All experimental procedures were conducted under the approval of the Ethics Committee of Southwest Medical University (License Number: 20220819–006).

### 2.7 Immunotoxicity assessment

The potential inflammatory and myelotoxic (neutropenia) effects of GO and PLL-modified titanium sheets were investigated using transgenic Tg (lyz:DsRed2) zebrafish embryos expressing red fluorescent protein (GFP) in neutrophils. This enabled direct observation of the impact of the study material on the emergence and accumulation of neutrophils in exposed tissues or internal organs. The transgenic zebrafish were bred to the adult stage following the feeding methods described above. Embryos were exposed to the test material at 36 hpf, then incubated at 28°C. At 120 hpf, the exposed embryos were imaged under a fluorescence microscope (Leica, Wetzlar, Germany), and the ImageJ program was used to assess the presence of neutrophils relative to the control group (based on fluorescence intensity). The experiment was performed twice, using five embryos in each group. The experimental procedures have been approved by the Ethics Committee of Southwest Medical University. Statistical analysis.

### 2.8 Statistical analysis

All data were presented as the mean ± standard deviation. Group comparisons were conducted using one-way ANOVA with Tukey’s test in GraphPad Prism (GraphPad Software, United States). Statistical significance was determined at *p < 0.05.

## 3 Results and discussion

### 3.1 Synthesis and morphology of materials

Implant restoration has greatly improved the quality of life of patients, and the growing sophistication of implantation technology has led to an increasing number of implant restorations. However, peri-implant disease remains a serious complication that jeopardizes the long-term survival of implants (Rokaya et al., 2020). This is due to the lack of real periodontal ligaments and blood supply around the implant, bacteria are more likely to invade the surrounding tissue, and inflammation progresses more rapidly (Herrera et al., 2023). The chemical composition of the implant surface can influence the adhesion of proteins and bacteria (Yu et al., 2022). Therefore, proper modification of titanium surface to inhibit bacterial adhesion and growth is a method worth considering (Suzuki et al., 2023).

**FIGURE 3:**
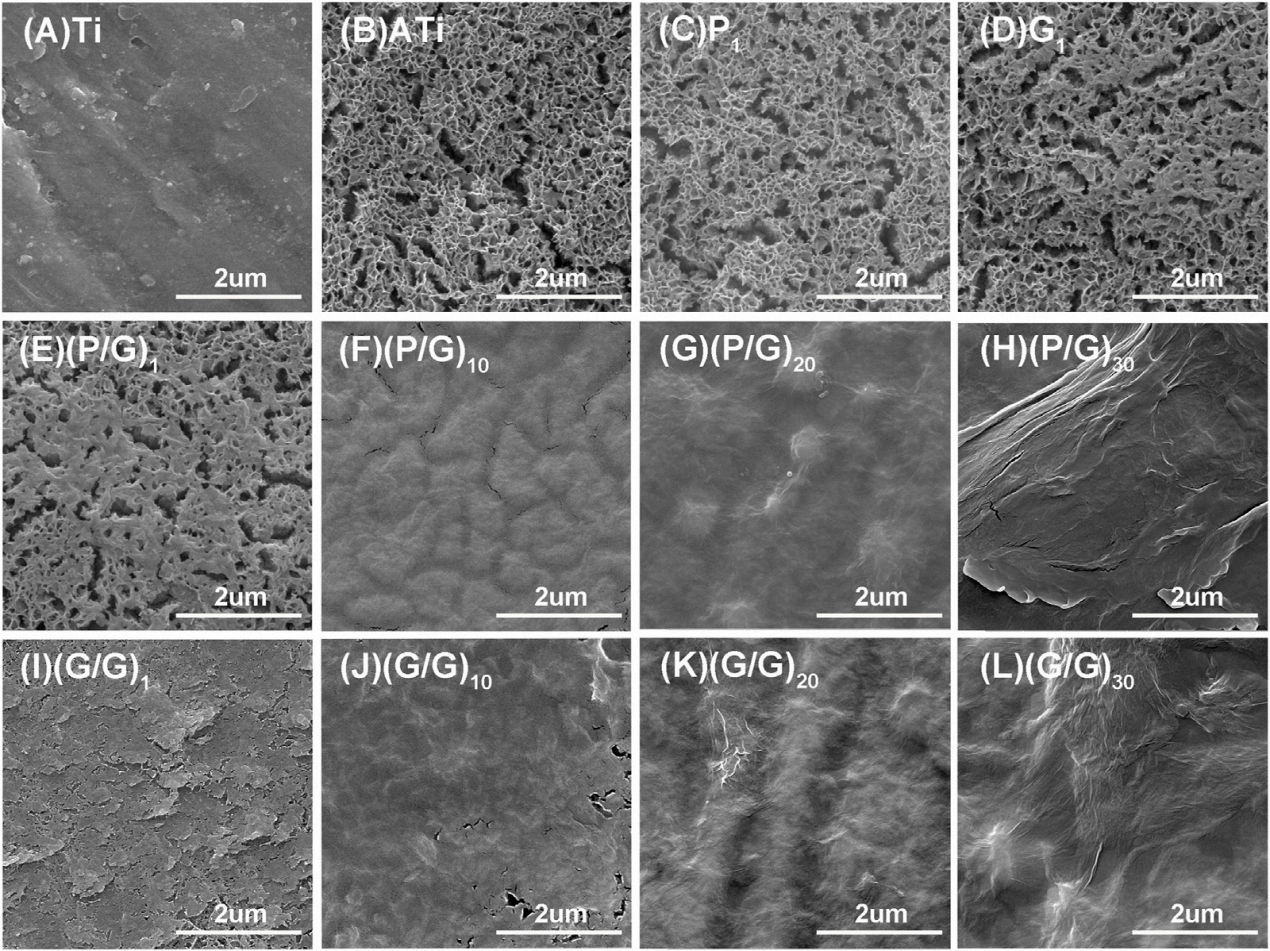
SEM morphology observation of titanium sheet surface. **(A)** The surface of pure Ti is smooth and dense. **(B)** After alkali activation, numerous mesh pores and cracks appear. **(C)** After depositing a layer of PLL, the surface of the titanium sheet is covered with extremely small PLL, presenting a foggy appearance. **(E)** After depositing a layer of P/G, some pores are blocked by the material. **(F) (G) (H)** After assembling 10, 20, and 30 layers of P/G, the pores and cracks are gradually filled and leveled. **(D)** After depositing a layer of GO-COOH, most of the pores are blocked. **(I–L)** After assembling 1, 10, 20, and 30 layers of G/G, the surface pores and cracks disappear with the increasing number of layers.

LBL technology has found extensive application in the functionalization of biomaterials. It has the capability to alter the surface properties of the substrate, thereby improving or enhancing tissue function. Recently, LBL technology has been introduced into the manufacturing of implant coatings (Gentile et al., 2015; Liu et al., 2022). PLL and GO-NH_2_ in aqueous solution are positively charged, while GO and GO-COOH are negatively charged, which makes it possible to deposit GO and PLL functional films on titanium surface by LBL technology (Guo et al., 2021). In order to make GO and PLL firmly combine on the surface of titanium sheet, firstly, the surface of titanium sheet was treated with 5 mol/L NaOH to make it full of negative charges (Kokubo and Yamaguchi, 2015). As depicted in Figure 3, the surface of pure titanium sheet is relatively smooth and dense, with the surface elements predominantly composed of titanium. After alkali activation treatment, the content of oxygen increased to 43.31%, the surface has a honeycomb grid structure and there are a lot of cracks, which was consistent with the phenomenon observed by He et al. (Kokubo and Yamaguchi, 2015; He et al., 2022; Jiang et al., 2023). After loading a layer of PLL, the bottom and surface of the fissure groove seem to be covered with fog, which is a very small PLL deposition. After completing the assembly of a PLL/GO, some cellular grids are blocked by materials. By alternately assembling different layers, the control of GO dosage is realized, which intuitively shows that with the increase of assembly layers, the pores and cracks on the surface of titanium sheet are gradually filled up. In Groups (P/G)_10_ and (G/G)_10_, although only a few cracks are not completely blocked by materials, the direction of the cracks at the bottom can be clearly seen. The surface gaps in the (P/G)_20_ and (G/G)_20_ groups were completely filled, while the surfaces of the (P/G)_30_ and (G/G)_30_ groups slightly protruded, exhibiting an appearance similar to that of crumpled paper. After PLL and GO are assembled, C and N elements begin to appear, C is provided by PLL and GO, and N comes from PLL. In the GO self-assembly groups, the presence of C and N elements indicates the successful assembly of GO-COOH and GO-NH_2_, where N comes from GO-NH_2_ (Table 1).

**TABLE 1:** Elemental compositions of the different samples.

Samples	Atomic concentration (at%)			
	C	N	O	Ti
Ti	-	-	-	100
ATi	-	-	43.31	56.69
(P/G)_10_	13.96	3.14	37.79	45.11
(P/G)_20_	15.55	2.86	40.07	41.52
(P/G)_30_	27.31	2.11	32.26	38.32
(G/G)_10_	18.46	2.37	35.97	43.2
(G/G)_20_	28.74	2.23	29.98	39.05
(G/G)_30_	37.95	0.8	27.67	33.58

### 3.2 FTIR

**FIGURE 4:**
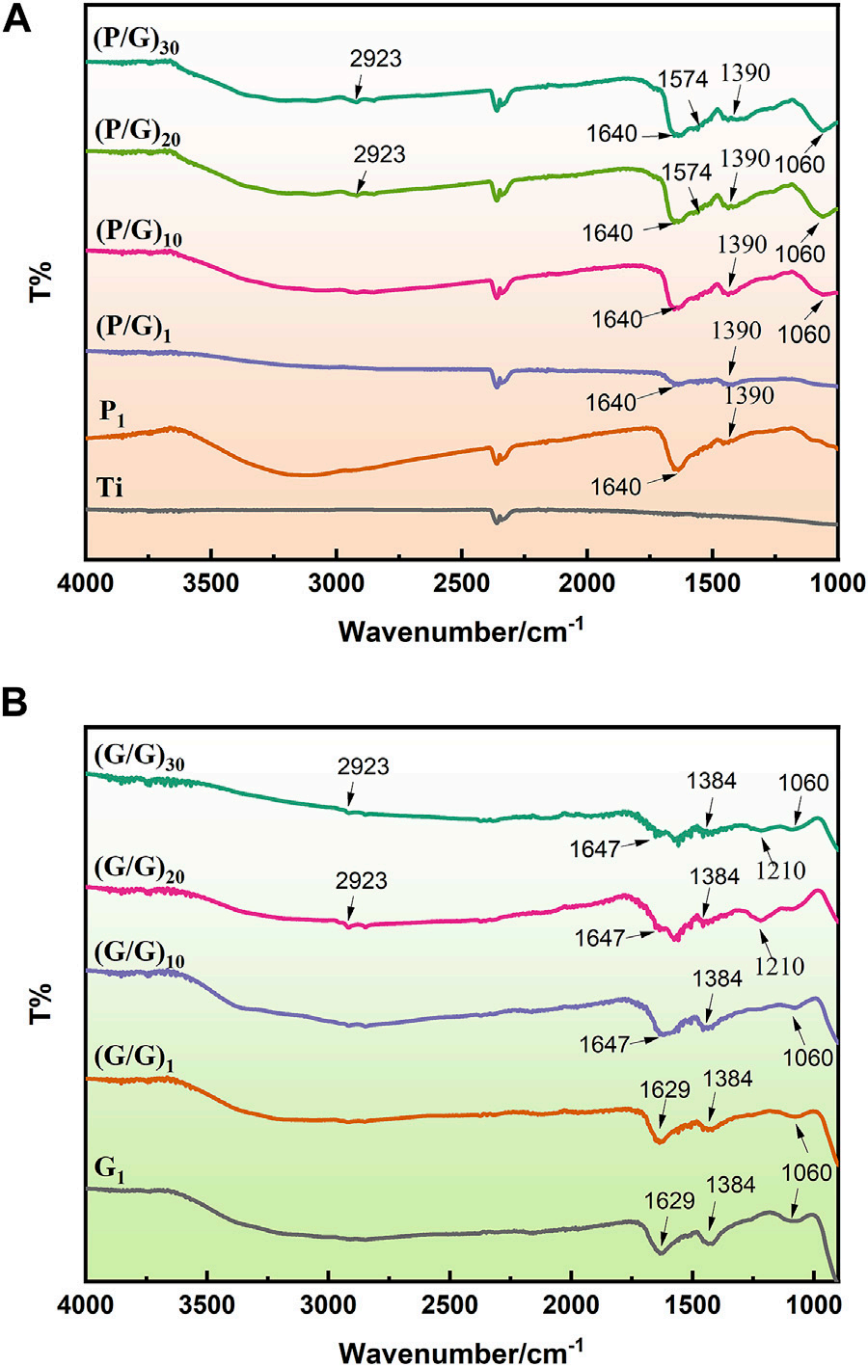
**(A)** FTIR spectra of the P/G group. **(B)** FTIR spectra of the G/G group.

The samples deposited with PLL exhibit a C=O stretching vibration peak at 1,640 cm^-1^, and an NH bending vibration peak at 2,923 cm^-1^, confirming the successful bonding of PLL to the sample surface (Guo et al., 2021). On the surface of the sample adsorbed with GO, characteristic peaks of oxygen-containing functional groups such as C=O (1,640 cm^-1^), C-OH (1,390 cm^-1^) and C-O-C (1,060 cm^-1^) appear, confirming the presence of GO coating on the modified material (Suo et al., 2019) (Figure 4). The sample assembled from GO-NH2 and GO-COOH shows characteristic peaks of GO, such as C-C (1,647 cm^-1^), C=C (cm^-1^), C-O-C (1,060 cm^-1^), and C-OH (1,390 cm^-1^). Additionally, characteristic peaks of carboxyl groups, C=O (1,640 cm^-1^), and amino groups, N-H (1,574 cm^-1^), as well as C-N (1,210 cm^-1^) peaks, are observed on the surface, indicating the success of the self-assembly. Moreover, with the number of assembly layers exceeding 10, the intensity of the C-O-C vibration peak of GO and the CH_2_ vibration peak of PLL also increase, indicating an increase in the relative content of PLL and GO (Figure 4A).

### 3.3 XPS analysis

**FIGURE 5:**
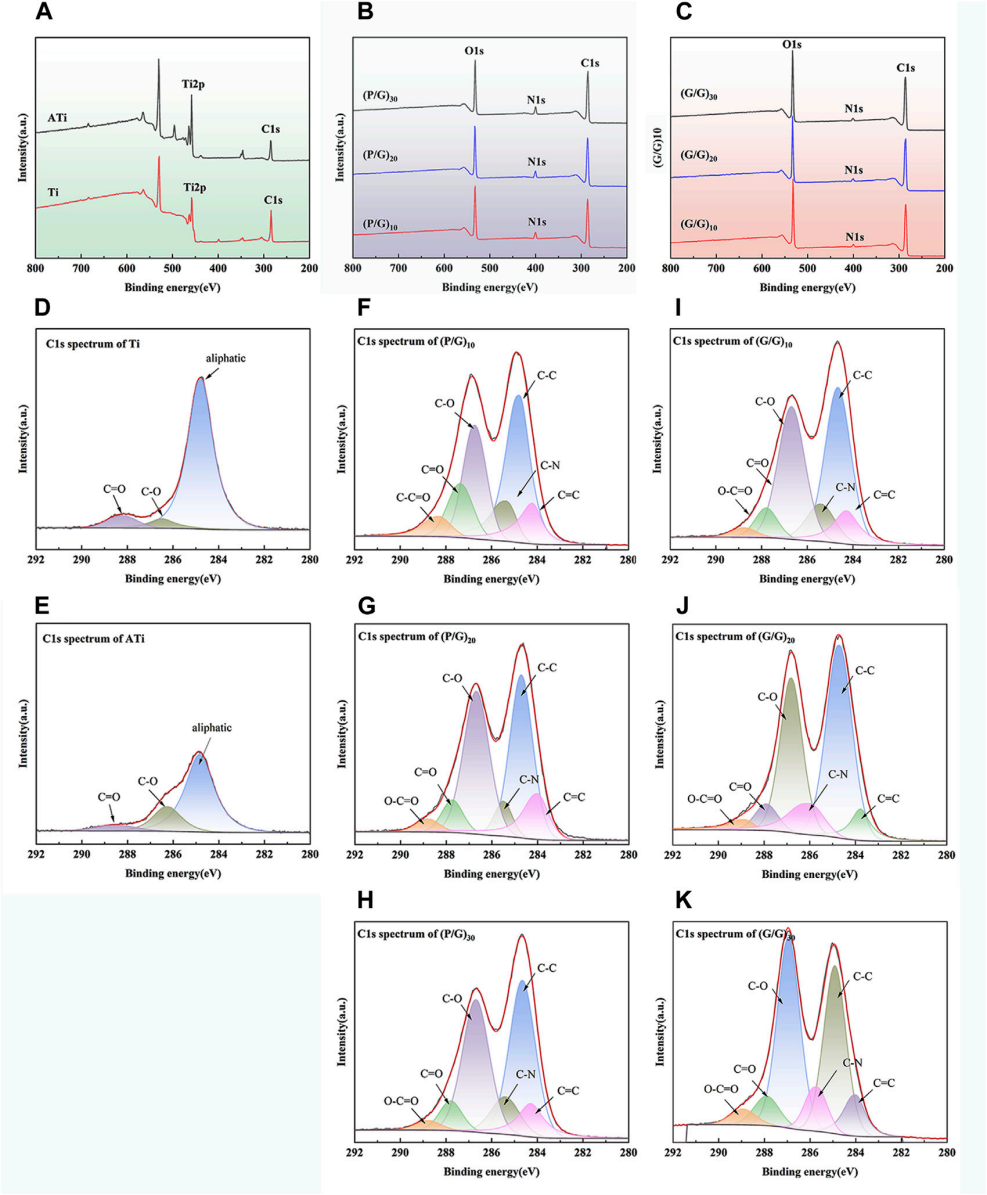
Surface chemical characteristics of different titanium samples: **(A)** XPS spectra of Ti and ATi surfaces; **(B)** XPS measurement spectra of (P/G)_10_, (P/G)_20_, (P/G)_30_ surfaces; **(C)** XPS measurement spectra of (G/G)_10_, (G/G)_20_, (G/G)_30_ surfaces; **(D**–**K)** High-resolution C1 peak fitting of surfaces of different titanium samples.

The surface chemical composition of samples was thoroughly analyzed using XPS technology. As shown in Figure 5A, three primary characteristic signals, Ti, C, and O, can be clearly identified in Ti and ATi samples, with the C and O signals originating from contaminant amino acids that were not completely removed from the sample surfaces. It is noteworthy that the XPS analysis results reveal that the O signal on the titanium surface increased after alkaline activation. Furthermore, on all samples treated with self-assembly, the characteristic Ti signal was no longer present, and only signals corresponding to C, N, and O were observed, with the C signal intensity being significantly higher than that on the Ti and ATi surfaces (Figures 5B,C). This phenomenon indicates that GO and PLL were uniformly deposited on the pure titanium surface, and its thickness exceeded the detection range of XPS. As shown in Figures 5F–I, samples such as (P/G)_10_, (P/G)_20_, (P/G)_30_, (G/G)_10_, (G/G)_20_, and (G/G)_30_ exhibited extremely similar high-resolution C1s spectra. The C1s spectrum contained six main peaks, corresponding to O-C=O (289.1 eV), C=O (287.9 eV), C-O (286.6 eV), C-N (285.5 eV), C=C (289.1 eV), and C-C (284.7 eV) chemical states. The nitrogen in these peaks originated from PLL and GO-NH2, while the carbon originated from PLL and GO-COOH.

### 3.4 Contact angle measurement

**FIGURE 6:**
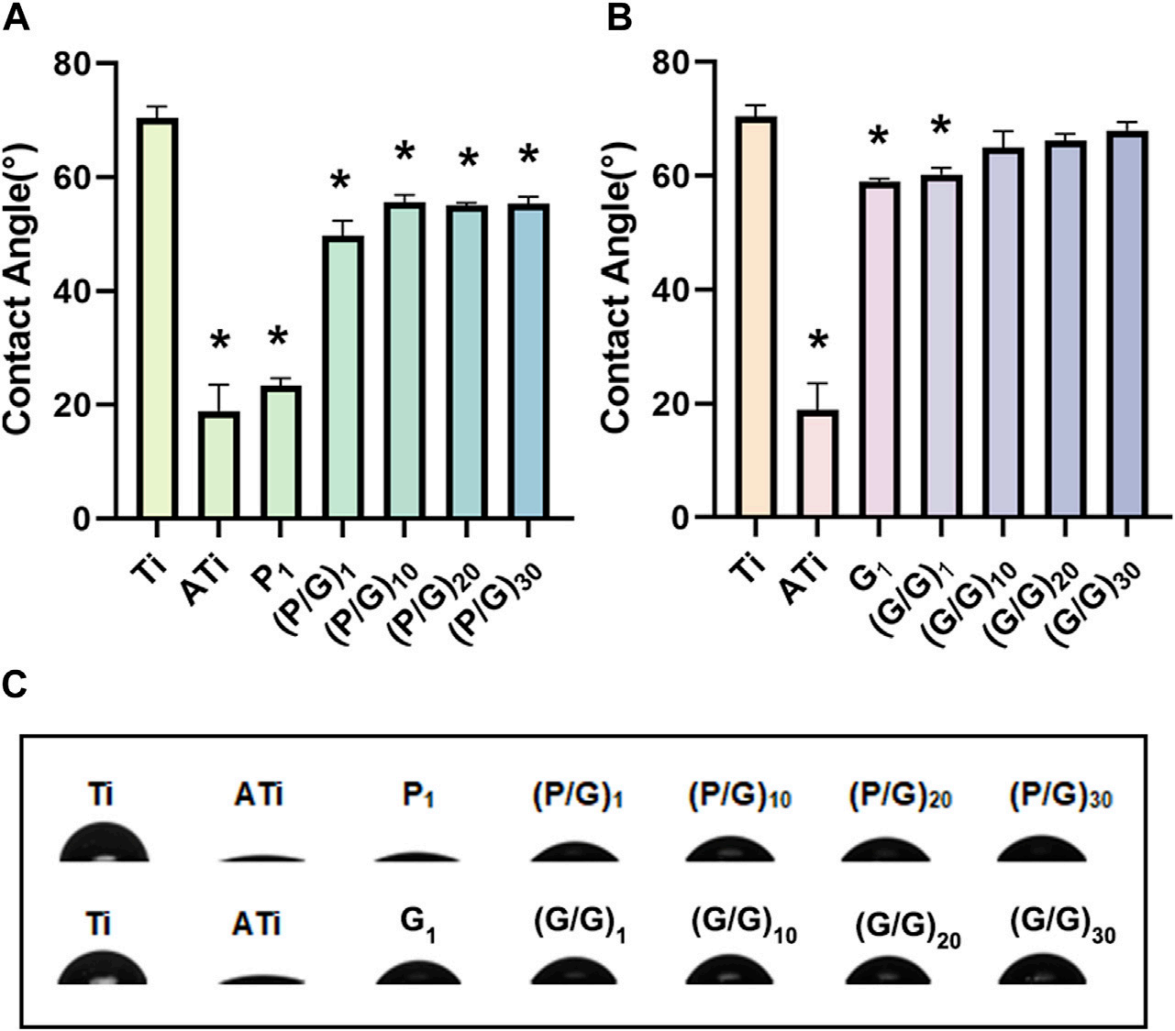
**(A)** Statistical analysis of water contact angles for the P/G group (n = 3). **(B)** Statistical analysis of water contact angles for the G/G group (n = 3). **(C)** Morphology of water droplets on different surfaces. (*p < 0.05).

The water contact angle on the surface of titanium sheet is approximately 70.47° ± 1.95°. After alkali activation, the surface becomes super-hydrophilic, with a contact angle of about 18.87° ± 4.7°, significantly different from that of the pure titanium surface (Figure 6A). This difference is attributed to the abundant hydroxyl (-OH) groups generated after alkali activation, interacting with water molecules to form hydrogen bonds, facilitating the spreading of water droplets on the titanium surface. With the layer-by-layer assembly of PLL and GO, the water contact angle gradually increases but remains significantly lower than that of pure titanium. We hypothesize that the hydroxyl groups on the titanium sheet surface are gradually consumed, and the van der Waals force produced by the interaction of carboxyl, amine, hydroxyl and other polar functional groups on the newly deposited PLL and GO surface is weak. Unfortunately, although a significant decrease in contact angle was observed when GO self-assembled a layer, the 10, 20 and 30 layers observed in the experiment all showed contact angles similar to those of pure titanium (Figure 6B). Studies have shown that hydrophilic surfaces with water contact angles less than 60° exhibit better cell binding and diffusion rates than hydrophobic surfaces (Jurak et al., 2021). Li et al. also pointed out that a rough hydrophilic titanium surface can reduce the production of pro-inflammatory factors by neutrophils (Abaricia et al., 2020). It can be observed that the biocompatibility of PLL and GO modified titanium tablets is better than that of pure titanium and GO modified titanium sheet alone, resulting in less tissue irritation and inflammation.

### 3.5 Antibacterial activity

#### 3.5.1 SEM morphology of bacteria

**FIGURE 7:**
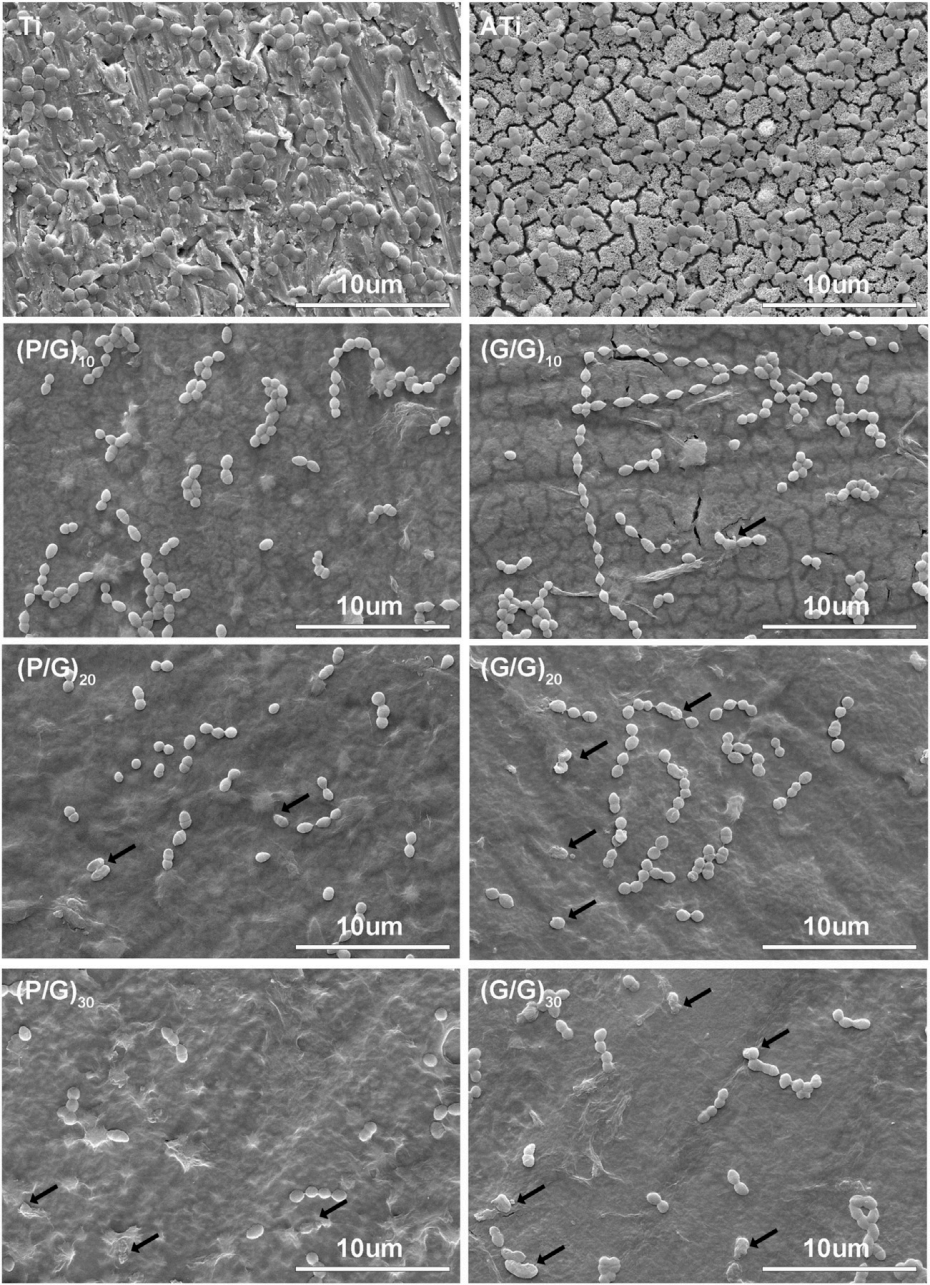
SEM image of bacteria. The black arrow shows ruptured and deformed bacteria.

*P.g* is considered to be the main periodontal pathogen associated with peri-implantitis (Pérez-Chaparro et al., 2016; Alves et al., 2022). After 24 h of co-cultivation with P.g, the number and morphology of bacteria on the titanium sheet surface were observed using SEM (Figures 6, 7). *P.g* on the surface of Ti and ATi titanium sheets grew well, covering almost the entire material surface, with smooth bacteria displaying neat edges and an oval shape. In both the P/G and G/G groups, it was observed that with the increase of loading layers, *P.g* grew from chain-like head-to-tail connection to sporadic form, and bacterial rupture and deformation became ubiquitous (shown by black arrows). Interestingly, compared to the P/G group with the same number of layers, the G/G group showed more bacterial rupture and deformation, although the number of bacteria was relatively higher. GO can produce ROS damage cellular components through oxidative stress, but it is in the state of suspension (Shi et al., 2016). In this experiment, GO tended to cause physical damage to the bacterial membrane by exerting membrane stress through its sharp edges.

#### 3.5.2 Inhibition zone test

**FIGURE 8:**
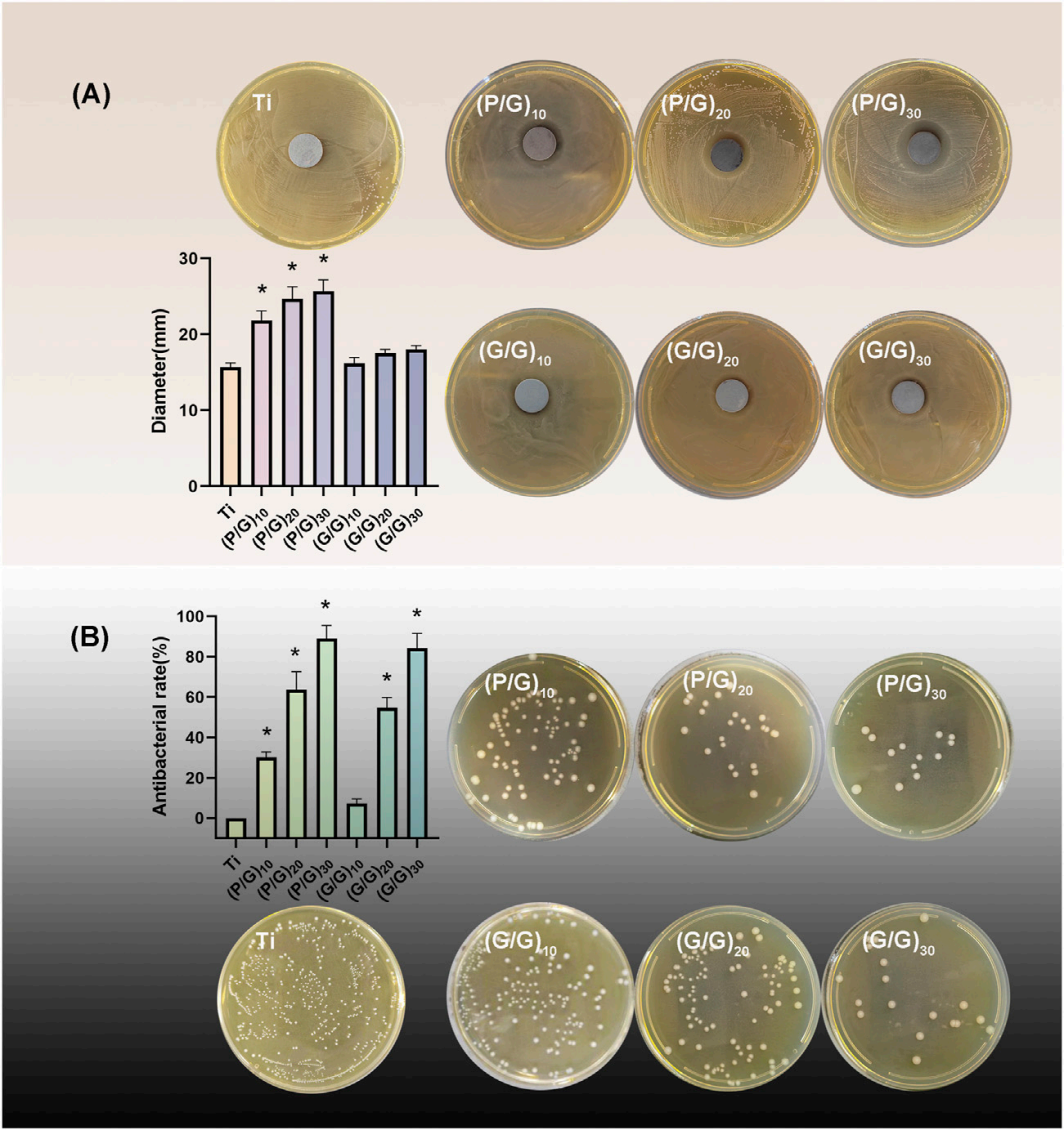
**(A)** Inhibition zone test and statistical analysis of each group (n = 3); **(B)**Colony count and inhibition rate statistics (n = 3). (*p < 0.05).

The ZOI method was employed to assess the inhibitory ability of the material on the growth of *P.g*. As shown in Figure 8A, only a tiny circular area free of bacteria was observed around the G/G group with all layers, showing no significant difference compared to the pure Ti group. On the contrary, obvious bacteriostatic ring appeared around the P/G modified titanium sheet, and the diameter of bacteriostatic ring increased with the increase of loading layers. This indicates that GO has difficulty diffusing from titanium sheet, while PLL can smoothly diffuse around the titanium sheet smoothly and effectively inhibit the growth of *P.g*. Literature has pointed out that PLL can not only act on cell wall and cell membrane system, but also target genetic materials, enzymes and functional proteins, causing disruptions in material, energy and information transmission within cells and ultimately leading to cell death (Wang et al., 2021).

#### 3.5.3 Antibacterial rate

The bacteriostatic rate of P.g was evaluated by plate counting method. The antibacterial rate of the control group was defined as 0, and the results revealed that the bacteriostatic rates of (P/G)_10_, (P/G)_20_, (P/G)_30_ were 30.1% ± 2.69%, 63.5% ± 8.92%, 88.99% ± 6.43%, (G/G)_10_, (G/G)_20_, (G/G)_30_ were 7.26% ± 2.33%, 54.5% ± 5.03%, 84.15% ± 7.44%, respectively (Figure 8B). GO has bacteriostatic ability either alone or in combination with PLL, but the bacteriostatic effect is limited when the content is low. At the same number of layers, the bacteriostatic ability of the group P/G was better than that of the group G/G, which indicated that the combined self-assembly of GO and PLL is a more effective antibacterial strategy. This approach can exert an antibacterial effect on the bacteria adhered to the implant surface as well as those suspended around the implant.

### 3.6 Cytotoxicity assays

**FIGURE 9:**
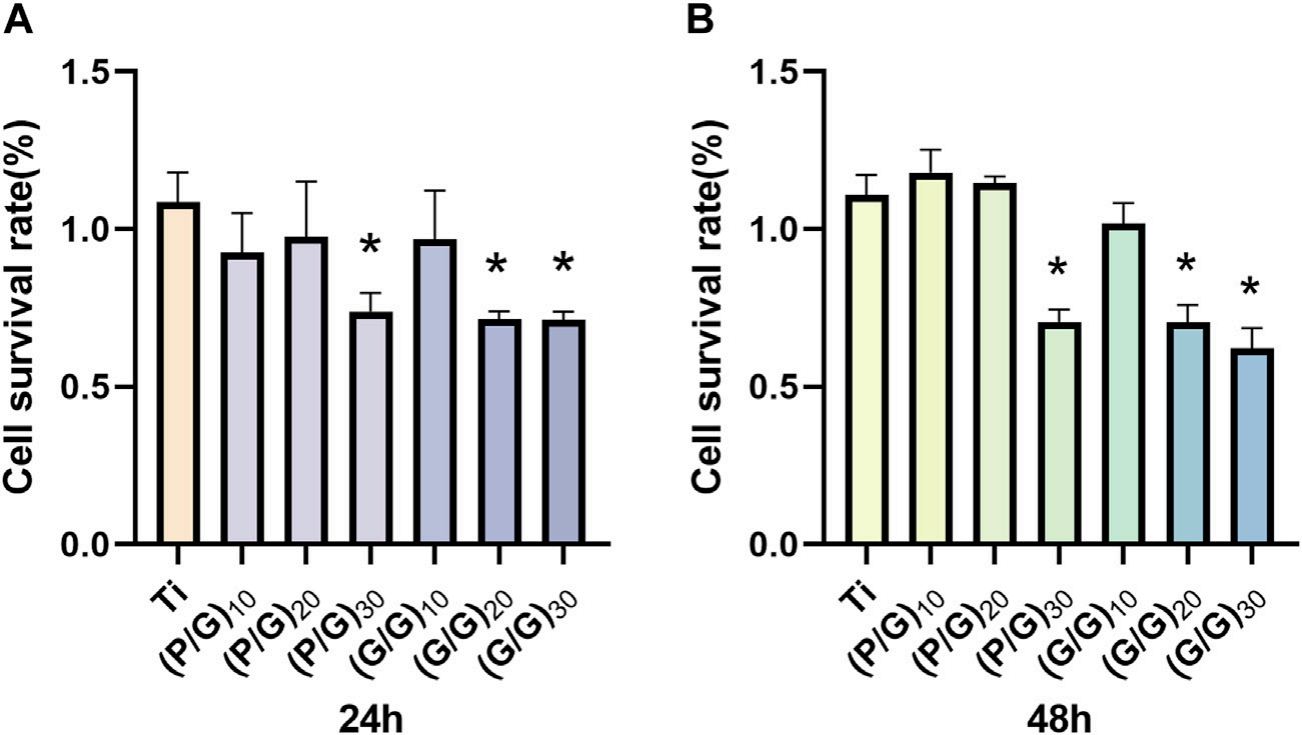
**(A)** The cell survival rate of MC3T3-E1 cells in each group at 24 h (n = 3). **(B)** The cell survival rate of MC3T3-E1 cells in each group at 48 h (n = 3). (*p < 0.05).

Osteoblasts play a crucial role in osseointegration. They adhere, spread, proliferate, and differentiate on the surface of implant materials, ultimately mineralizing into bone and forming a solid union with the bone implants. The murine-derived osteoblast cell line MC3T3-E1 is commonly employed for studying the biocompatibility of bone implants (Czekanska et al., 2012). As can be seen from Figure 9, with an increase in the number of film layers, the cell survival rate of the experimental group showed a decreasing trend compared with the control group. The cell viability of groups (P/G)_20_, (G/G)_20_ and (G/G)_30_ showed a significant decrease at both 24 h and 48 h. A large number of studies (Pang et al., 2017; Pulingam et al., 2020) have shown that cytotoxicity is positively correlated with the concentration of graphene oxide. This is because PLL itself can be degraded into essential amino acids for the human body, suggesting that cytotoxicity comes from GO and is concentration-dependent. Moreover, as long as the assembly of PLL and GO does not reach 30 layers, it remains relatively safe for MC3T3-E1 cells and can be used in oral implant repair.

### 3.7 *In vivo* toxicity assessment

**FIGURE 10:**
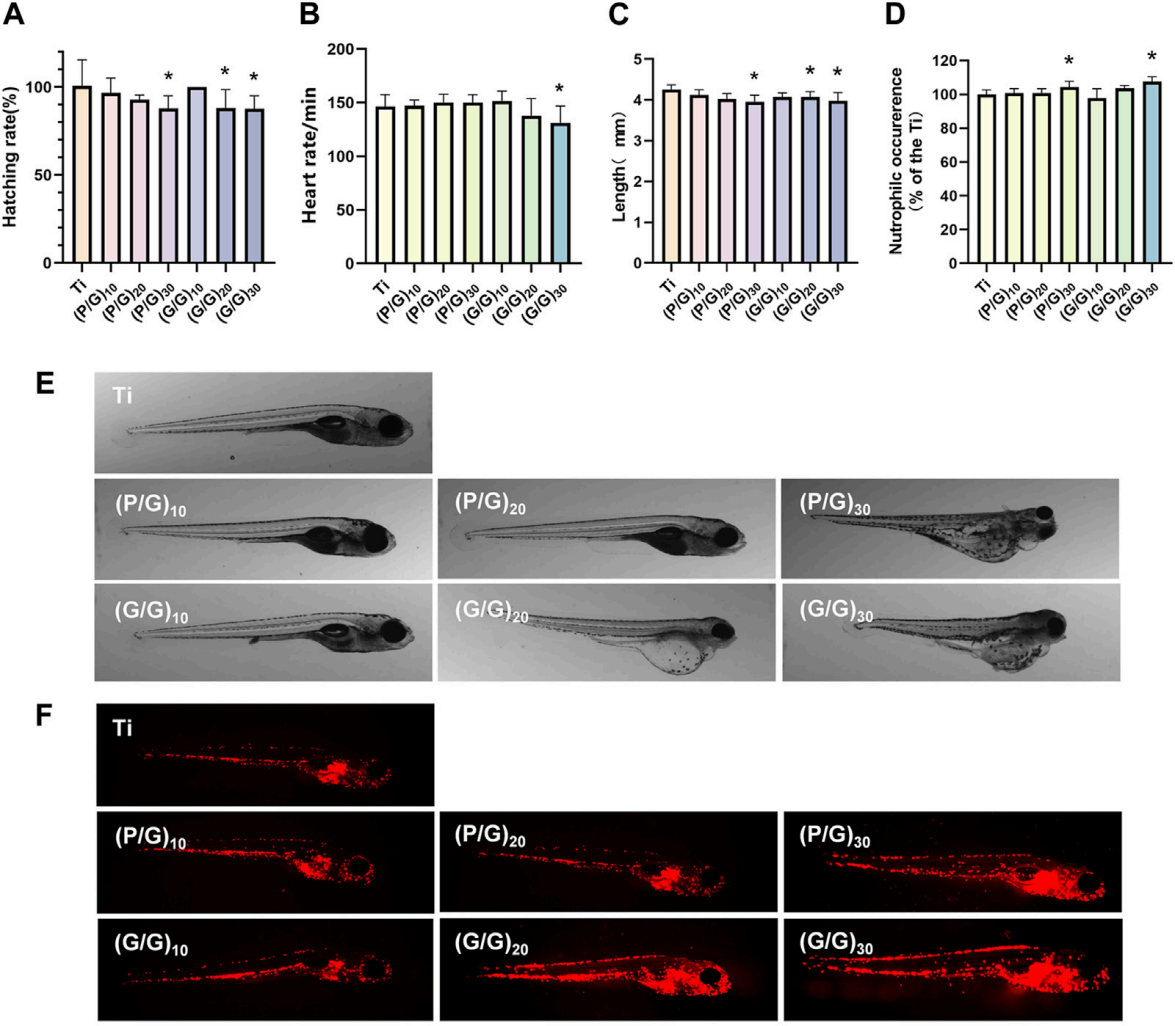
**(A)** Zebrafish embryo hatching rate (n = 3). **(B)** Zebrafish heart rate (n = 10). **(C)** Body length of zebrafish in each group (n = 15). **(D)**growth and development morphology of zebrafish in each group. **(E)** Analysis of neutrophil fluorescence intensity in transgenic zebrafish. **(F)** Appearance of neutrophils under fluorescence microscope (n = 10). (*p < 0.05).

Zebrafish have up to 70% genomic homology compared to the human genome (Howe et al., 2013; Miyawaki, 2020). Moreover, zebrafish possess excellent characteristics such as *in vitro* fertilization and development, embryo transparency, and easy breeding. Hence, it stands out as an exceptional model organism for evaluating the biocompatibility and therapeutic efficacy of new biological materials (Feng et al., 2022). When wild type (AB) zebrafish were co-cultured with titanium sheets during the highly material sensitive early embryonic stage (6 hpf), there was no significant increase in embryo mortality in all experimental groups compared with the control group. Under normal conditions, most embryos would break through the chorions to complete incubation at 72 hpf, however, there was a delay in hatching (Figure 10A) observed in the (P/G)_30_, (G/G)_20_, and (G/G)_30_ group. This is consistent with the results of Chen et al., they confirmed that GO will adsorb on the surface of the chorion, blocking the pores between the chorionic processes, and gradually form an anoxic environment in the embryo, which leads to the delay of hatching (Chen et al., 2020). In addition, studies have shown that GO induces edema in the pericardium and yolk sac, which disrupts the activity of hatching enzymes and may be another reason for delayed hatching (Chen et al., 2016). The heart is the earliest organ that occurs and functions in the process of embryonic development, and the heart rate is the most intuitive reaction of the normal function of the heart (Chen et al., 2020). However, when the embryos developed to 120 hpf, the heart rate decreased in (G/G)_30_ groups (Figure 10B), the body surface of zebrafish in (G/P)_30_, (G/G)_20_, (G/G)_30_ groups was no longer smooth (Figure 10E), There seems to be floc shedding and adhesion, and there are many developmental deformities, including pericardial edema, yolk sac edema, and tail deformity. In addition, a reduction in body length was observed in the (P/G)_30_, (G/G)_20_, and (G/G)_30_ group (Figure 10C). In the study by Chen et al., zebrafish embryos exposed to 1 mg/L GO also showed a significant reduction in heart rate (Chen et al., 2016). This suggests that high concentrations of GO may produce certain cardiotoxicity to zebrafish, which in turn affects growth and development.

### 3.8 Immunotoxicity assessment

Taking advantage of the transparent nature of zebrafish embryos, the neutrophils of transgenic zebrafish Tg (lyz:DsRed2) are labeled red by fluorescent proteins, which can be used to evaluate the early inflammatory response and immunotoxicity that we are very concerned about when planting. After 120 hpf direct exposure, (P/G)_30_ and (G/G)_30_ groups showed a significant increase in neutrophils (Figure 10D), and the fluorescence was mainly concentrated in the yolk sac, heart and periocular area (Figure 10F). It has been reported that once GO enters the embryo, it will selectively locate the yolk sac, heart, eye and tail, affecting the early development of the embryo, and the incidence of early malformation increases with the increase of GO concentration.

## 4 Conclusion

This study successfully performed separate assemblies of 10, 20, and 30 layers of GO on the titanium surface, as well as combined assemblies of GO and PLL. The results indicate that self-assembly of GO with more than 10 layers exhibits good antibacterial performance, and the joint assembly of GO and PLL demonstrates superior antibacterial efficacy under the same layer conditions. Additionally, assemblies of P/G with less than 30 layers and G/G with less than 20 layers exhibit safe biological properties, without causing cytotoxicity, developmental toxicity, and immunotoxicity. Considering the balance between antibacterial performance and biological toxicity, the combined assembly of GO and PLL with 20 layers presents a promising choice for designing and optimizing titanium-based implants.

## Data Availability

The raw data supporting the conclusions of this article will be made available by the authors, without undue reservation.
