# Supplementary material for: Graphene oxide/ε-poly-L-lysine self-assembled functionalized coatings improve the biocompatibility and antibacterial properties of titanium implants
Source: Front Bioeng Biotechnol. 2024 Apr 4;12:1381685. doi: 10.3389/fbioe.2024.1381685 (PMC11024266; doi:10.3389/fbioe.2024.1381685)
Supplement: Supplementary file 2 [file DataSheet1.docx]

Supplementary Material

**Graphene Oxide/ε-Poly-L-Lysine Self-assembled Functionalized**

**Coatings Improve the Biocompatibility and Antibacterial**

**Properties of Titanium Implants**

**Xiaoxiao You, Zhongke Wang, Li wang, Youbo Liu, Xiaorong Lan, Ling Guo***

*** Correspondence:** Ling Guo (gl2005202@foxmail.com)


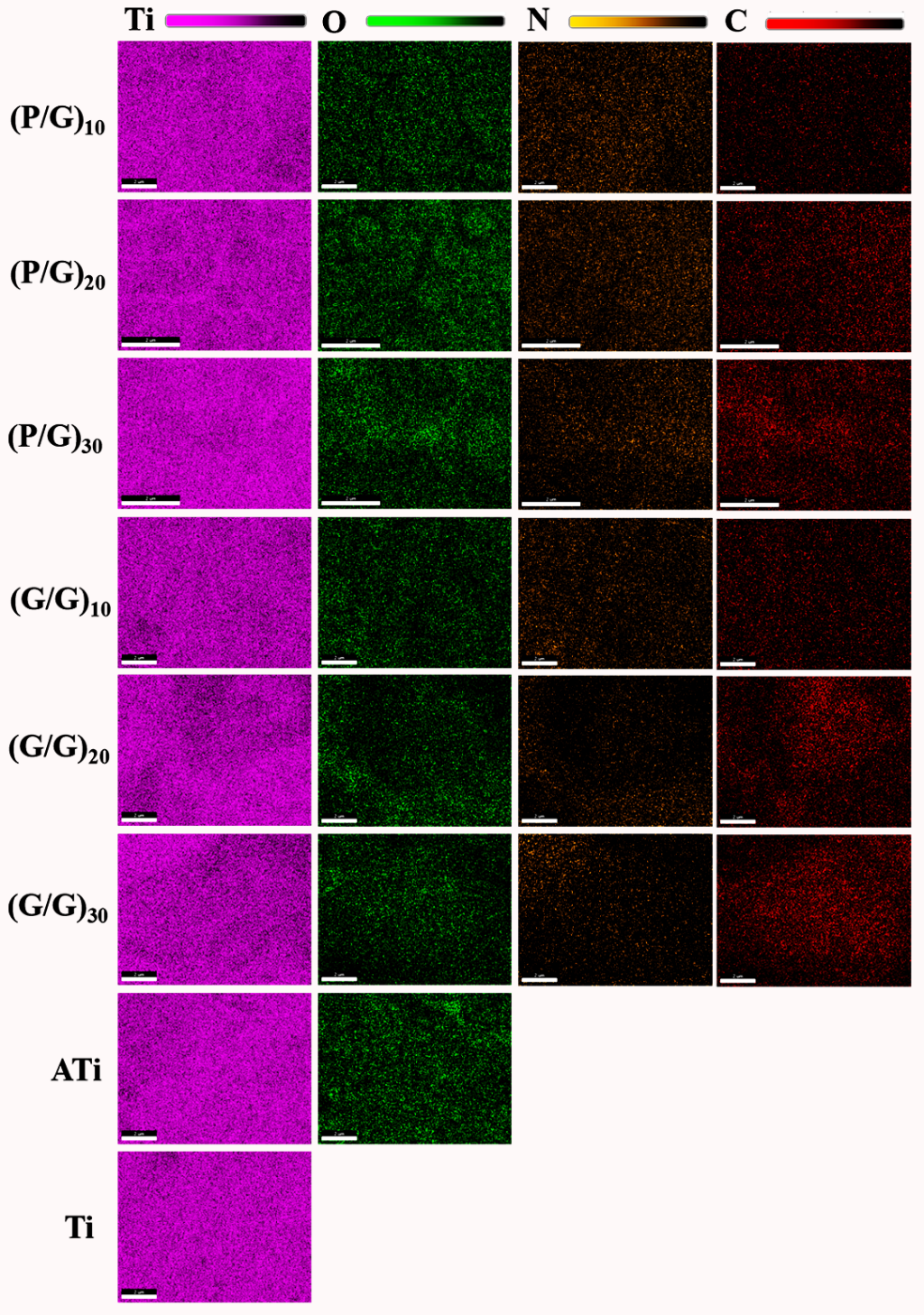


***Figure S1.*** The mapping images of different sample surfaces by X-ray energy dispersive spectrometer (EDS) showed that the (P/G)_10_, (P/G)_20_, (P/G)_30_, (G/G)_10_, (G/G)_20_, (G/G)_30_ all contained Ti,O,N and C elements, and the distribution of the four elements was relatively uniform. With the increase of the number of assembly layers, the content of O, N and C elements on the surface of the sample increased slightly, while the content of Ti decreased due to the coating.

**
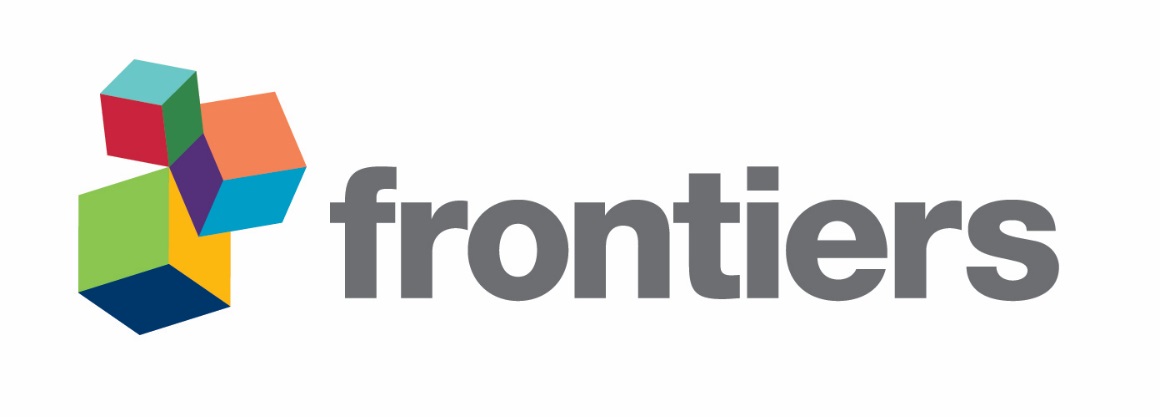
**
